# A prediction rule for severe adverse events in all inpatients with community-acquired pneumonia: a multicenter observational study

**DOI:** 10.1186/s12890-022-01819-0

**Published:** 2022-01-12

**Authors:** Toshihiro Sakakibara, Yuichiro Shindo, Daisuke Kobayashi, Masahiro Sano, Junya Okumura, Yasushi Murakami, Kunihiko Takahashi, Shigeyuki Matsui, Tetsuya Yagi, Hideo Saka, Yoshinori Hasegawa

**Affiliations:** 1 Department of Respiratory Medicine, Nagoya University Graduate School of Medicine, 65 Tsurumai-cho, Showa-ku, Nagoya, 466-8550 Japan; 2 Kyoto University Health Service, Kyoto, Japan; 3 Department of Respiratory Medicine, Higashi Nagoya National Hospital, Nagoya, Japan; 4 Department of Respiratory Medicine, Toyota Memorial Hospital, Toyota, Japan; 5 Department of Biostatistics, Nagoya University Graduate School of Medicine, Nagoya, Japan; 6 Department of Biostatistics, M&D Data Science Center, Tokyo Medical and Dental University, Tokyo, Japan; 7 Department of Infectious Diseases, Nagoya University Hospital, Nagoya, Japan; 8 Department of Respiratory Medicine, Matsunami General Hospital, Gifu, Japan; 9 National Hospital Organization, Nagoya Medical Center, Nagoya, Japan

**Keywords:** Severe pneumonia, Severity, Mortality, Healthcare-associated pneumonia, Prediction score

## Abstract

**Background:**

Prediction of inpatients with community-acquired pneumonia (CAP) at high risk for severe adverse events (SAEs) requiring higher-intensity treatment is critical. However, evidence regarding prediction rules applicable to all patients with CAP including those with healthcare-associated pneumonia (HCAP) is limited. The objective of this study is to develop and validate a new prediction system for SAEs in inpatients with CAP.

**Methods:**

Logistic regression analysis was performed in 1334 inpatients of a prospective multicenter study to develop a multivariate model predicting SAEs (death, requirement of mechanical ventilation, and vasopressor support within 30 days after diagnosis). The developed ALL-COP-SCORE rule based on the multivariate model was validated in 643 inpatients in another prospective multicenter study.

**Results:**

The ALL-COP SCORE rule included albumin (< 2 g/dL, 2 points; 2–3 g/dL, 1 point), white blood cell (< 4000 cells/μL, 3 points), chronic lung disease (1 point), confusion (2 points), PaO_2_/F_I_O_2_ ratio (< 200 mmHg, 3 points; 200–300 mmHg, 1 point), potassium (≥ 5.0 mEq/L, 2 points), arterial pH (< 7.35, 2 points), systolic blood pressure (< 90 mmHg, 2 points), PaCO_2_ (> 45 mmHg, 2 points), HCO_3_^−^ (< 20 mmol/L, 1 point), respiratory rate (≥ 30 breaths/min, 1 point), pleural effusion (1 point), and extent of chest radiographical infiltration in unilateral lung (> 2/3, 2 points; 1/2–2/3, 1 point). Patients with 4–5, 6–7, and ≥ 8 points had 17%, 35%, and 52% increase in the probability of SAEs, respectively, whereas the probability of SAEs was 3% in patients with ≤ 3 points. The ALL-COP SCORE rule exhibited a higher area under the receiver operating characteristic curve (0.85) compared with the other predictive models, and an ALL-COP SCORE threshold of ≥ 4 points exhibited 92% sensitivity and 60% specificity.

**Conclusions:**

ALL-COP SCORE rule can be useful to predict SAEs and aid in decision-making on treatment intensity for all inpatients with CAP including those with HCAP. Higher-intensity treatment should be considered in patients with CAP and an ALL-COP SCORE threshold of ≥ 4 points.

***Trial registration*:**

This study was registered with the University Medical Information Network in Japan, registration numbers UMIN000003306 and UMIN000009837.

**Supplementary Information:**

The online version contains supplementary material available at 10.1186/s12890-022-01819-0.

## Background

Site-of-care decision according to disease severity is one of the most critical steps in the management of pneumonia [1]. Several established prediction models have been developed to identify patients with community-acquired pneumonia (CAP) who are at low risk for death and can be treated in an outpatient setting. The representative models include the Pneumonia Severity Index (PSI) and the CURB-65 (confusion, blood urea nitrogen > 7 mmol/L [20 mg/dL], respiratory rate ≥ 30/min, low blood pressure [diastolic blood pressure ≤ 60 mm Hg or systolic blood pressure < 90 mm Hg], and age ≥ 65 years) [2, 3].

Identification of patients at high risk for severe adverse events (SAEs) at the time of pneumonia diagnosis is also crucial. These risk assessments aid physicians in determining patients who require higher-intensity treatment [4]. Some of the prediction models proposed for the identification of these patients include the 2007 Infectious Diseases Society of America (IDSA)/American Thoracic Society (ATS) criteria [1], the España SCAP rule [5], and the SMART-COP [6]. However, approaches to identify and assess patients who might need more intense treatment compared to those at low risk for SAEs remain a debatable issue [7–9]. Furthermore, although the management of patients with CAP and those with healthcare-associated pneumonia (HCAP) have been considered as the same entity in the 2019 ATS/IDSA CAP guidelines [4, 10], evidence is limited regarding prediction models that can be applicable to all patients with CAP including those with HCAP to identify those who require higher-intensity treatment at the time of pneumonia diagnosis.

Therefore, we aimed to develop and validate a prediction tool for the identification of all patients with CAP who are at high risk for SAEs using two different cohort datasets that were prospectively collected.

## Methods

### Study design and setting

This study was performed using datasets of two different prospective observational studies [11, 12]. A prediction model was developed using a larger dataset from an observational study (derivation cohort), which was performed at ten medical institutions in Japan (one 1000-bed university hospital and nine major community hospitals, each equipped with more than 500 beds) between March and December 2010 [11, 13, 14]. The prediction model was validated using a dataset from another observational study (validation cohort), which was performed at four medical institutions between April 2013 and March 2014. These four institutions participated in the first study for the derivation cohort as well [12]. There were no overlapping cases between the derivation and validation cohorts.

The study protocol adhered to the Declaration of Helsinki and the Japanese Ethics Guidelines for Epidemiological Studies. The study was approved by the ethics committee of Nagoya University and respective institutional review boards of the participating institutions. Informed consent of the participants was waived, but the opt-out method was adopted according to the ethics guidelines.

### Patients

Details on pneumonia definitions and categories, inclusion and exclusion criteria, definitions of variables, procedures, and data collection have been described elsewhere [11, 12] and can be found in the Additional file 1. Briefly, patients aged 20 years or older who developed pneumonia outside of a hospital and needed inpatient treatment were enrolled. Definitions of pneumonia categories were in accordance with international guidelines [1, 15]. The study included adult patients with CAP including those with HCAP. Outpatients and patients with hospital-acquired and ventilator-associated pneumonia were excluded.

### Endpoints

The primary study endpoint was SAEs, which were composite and included all-cause death and requirement of mechanical ventilation (MV) or vasopressor support (VS) within 30 days after pneumonia diagnosis. MV included invasive and noninvasive MV. Secondary endpoints were individual components of the primary endpoint and intensive care unit (ICU) admission within 30 days after the pneumonia diagnosis.

### Model development

Patient data without missing values were used for model development (complete-case analysis). A total of 33 candidate variables potentially associated with SAEs, except type of pneumonia, were identified from the literature review and are shown in Additional file 2 and Table 1 [1–3, 5, 6, 16–18]. Categorization of continuous variables and cutoff values were determined with reference to previous studies [1–3, 5, 6, 16–18], clinical significance, and data distribution of each variable. First, univariate logistic regression analysis was performed to assess the effects of candidate variables on SAEs. Next, multivariate logistic regression analysis was performed using all 33 candidate variables. A forward stepwise selection procedure was used with the inclusion of variables with a *p* value of less than 0.1. β-coefficients, odds ratios (ORs), and the corresponding 95% confidence intervals (CIs) were calculated. A simple prediction scoring system was developed based on the multivariate logistic regression model by rounding β-coefficients. The frequencies of SAEs were calculated by points. Receiver operating characteristic (ROC) curves of the multivariate logistic regression model and the simple prediction scoring system were assessed. The point with higher sensitivity was preferred when determining the threshold of the simple prediction scoring system. We also considered that the possible acceptable specificity was at least 60% (between 60 and 70%) according to previous studies on the España SCAP rule and SMART-COP [4–6]. In addition, the ROC curve and calculated Youden’s index were utilized to determine the threshold [19].

**Table 1:** Patient characteristics and clinical outcomes

Variables	Derivation cohort	Validation cohort
	(n = 1334)	(n = 643)
*Age, years*		
< 65	250 (18.7)	106 (16.5)
65–79	528 (39.6)	269 (41.8)
≥ 80	556 (41.7)	268 (41.7)
Sex, female	463 (34.7)	208 (32.3)
*Pneumonia type*		
CAP^†^	836 (62.7)	437 (68.0)
HCAP^‡^	498 (37.3)	206 (32.0)
Nursing home resident	216 (16.2)	108 (16.8)
Nonambulatory status	327 (24.5)	133 (20.7)
Nonambulatory status or age ≥ 80 years	683 (51.2)	301 (46.8)
Heavy alcohol use^§^	40 (3.0)	19 (3.0)
*Comorbidities*		
Neoplastic diseases	191 (14.3)	93 (14.5)
Congestive heart failure	177 (13.3)	112 (17.4)
Chronic liver diseases	51 (3.8)	16 (2.5)
Cerebrovascular diseases	291 (21.8)	100 (15.6)
Diabetes	251 (18.8)	110 (17.1)
Immunosuppression^ll^	118 (8.8)	58 (9.0)
Chronic renal diseases	108 (8.1)	47 (7.3)
Chronic lung diseases	451 (33.8)	232 (36.1)
*Physical examination*		
Altered mental status (confusion)	266 (19.9)	142 (22.1)
Pulse rate ≥ 125/min	135 (10.1)	72 (11.2)
Body temperature < 36.0 °C	42 (3.1)	10 (1.6)
Systolic blood pressure < 90 mmHg	74 (5.5)	31 (4.8)
Respiratory rate ≥ 30/min	311 (23.3)	146 (22.7)
*Laboratory test*		
Blood urea nitrogen ≥ 30 mg/dL	298 (22.3)	132 (20.5)
Glucose, mg/dL		
< 70	19 (1.4)	9 (1.4)
≥ 250	97 (7.3)	39 (6.1)
Albumin, g/dL		
< 2.0	35 (2.6)	20 (3.1)
2.0–3.0	416 (31.2)	175 (27.2)
Sodium, mEq/L		
< 130	91 (6.8)	61 (9.5)
≥ 146	49 (3.7)	14 (2.2)
Potassium ≥ 5.0 mEq/L	122 (9.1)	38 (5.9)
Total bilirubin ≥ 2.0 mg/dL	59 (4.4)	19 (3.0)
White blood cell count < 4000 cells/μL	44 (3.3)	19 (3.0)
Hematocrit < 30%	192 (14.4)	72 (11.2)
Platelet count < 100 000 cells/μL	58 (4.3)	23 (3.6)
*Blood gas analysis*		
PaO_2_/F_I_O_2_ ratio		
≤ 100 mmHg	111 (8.3)	39 (6.1)
100–200 mmHg	209 (15.7)	90 (14.0)
200–300 mmHg	482 (36.1)	261 (40.6)
Arterial pH < 7.35	148 (11.1)	58 (9.0)
PaCO_2_ > 45 mmHg	212 (15.9)	77 (12.0)
HCO_3_^−^ < 20 mmol/L	170 (12.7)	63 (9.8)
*Radiological findings*		
Pleural effusion	324 (24.3)	133 (20.7)
Extent of total infiltration		
1/3–2/3 of unilateral lung	415 (31.1)	208 (32.3)
> 2/3 of unilateral lung	268 (20.1)	143 (22.2)
*Outcomes*		
Severe adverse events**	277 (20.8)	120 (18.7)
30-Day mortality	163 (12.2)	60 (9.3)
Requirement of MV or VS within 30 days^††^	154 (11.5)	74 (11.5)
Requirement of MV within 30 days	130 (9.7)	64 (10.0)
Requirement of VS within 30 days	83 (6.2)	28 (4.4)
ICU admission within 30 days	99 (7.4)	66 (10.3)

^*^Data are presented as no (%)

Definition of abbreviations: CAP, community-acquired pneumonia; HCAP, healthcare-associated pneumonia; MV, mechanical ventilation; VS, vasopressor support; ICU, intensive care unit

^†^CAP excluding HCAP was defined as pneumonia that did not match the criteria for hospital-acquired pneumonia (pneumonia occurring 48 h or more after hospital admission) or HCAP

^‡^HCAP was defined as pneumonia co-occurring with any of the following conditions: hospitalization for 2 days or more during the preceding 90 days, residence in a nursing home or extended care facility, at-home intravenous therapy (including antibiotics and chemotherapy), chronic dialysis (including hemodialysis and peritoneal dialysis) during the preceding 30 days, or home wound care during the preceding 30 days

^§^Heavy alcohol use was defined as a mean daily alcohol intake of 120 g /day

^ll^Immunosuppression included any immunosuppressive diseases, such as congenital or acquired immunodeficiency, hematologic diseases, asplenia and neutropenia (< 1000 cells/μL), treatment with immunosuppressive agents, chemotherapy within the previous 30 days, and corticosteroids in daily doses of at least 10 mg/day prednisone equivalent for more than 2 weeks

^**^Severe adverse events included death and requirement of mechanical ventilation (invasive or noninvasive) or vasopressor support within 30 days after pneumonia diagnosis

^††^38 patients in the derivation cohort and 14 patients in the validation cohort required invasive MV or VS at admission

### Model performance and validation

Sensitivity, specificity, positive predictive value (PPV), negative predictive value (NPV), area under the ROC curve (AUROC), and corresponding 95% CIs were calculated to assess model performance. Positive likelihood ratio (PLR) and negative likelihood ratio (NLR) were also calculated. These values were compared among prediction models using the validation cohort. Validation analyses were performed in patients without missing values.

### Severity assessment tools for comparisons

The PSI [2], CURB-65 [3], 2007 IDSA/ATS criteria [1], simplified 2007 IDSA/ATS minor criteria [20], SMART-COP [6], and España SCAP rule [5] were assessed for comparisons. For each prediction method, the diagnostic accuracy was assessed using the following thresholds reported in the original articles on these tools: PSI, ≥ class IV; CURB-65, ≥ 3 points; España SCAP rule, ≥ 10 points; SMART-COP, ≥ 3 points; 2007 IDSA/ATS criteria (minor criteria), ≥ 3 factors [21]; and simplified 2007 IDSA/ATS minor criteria, ≥ 3 factors [20]. During calculations for the 2007 IDSA/ATS criteria, simplified 2007 IDSA/ATS minor criteria, SMART-COP, and España SCAP rule, multilobar radiological infiltration was substituted by bilateral or chest radiographical infiltration extending to more than 2/3 of the unilateral lung.

### Subgroup analyses

Subgroup analyses were performed in patients without potential treatment restrictions, those with CAP (excluding HCAP) or HCAP, those without invasive MV/VS at admission, and those without immunosuppression. Patients without potential treatment restriction were defined as ambulatory patients under 80 years. Those with potential treatment restrictions were defined as those aged 80 years or older or any patient with nonambulatory status [22]. Invasive MV/VS at admission corresponded to the major criteria of the 2007 IDSA/ATS criteria [1]. Immunosuppression included any immunosuppressive diseases, such as congenital or acquired immunodeficiency, hematologic diseases, asplenia and neutropenia (< 1000 cells/μL), treatment with immunosuppressive agents, chemotherapy within the previous 30 days, and use of corticosteroids in daily doses of at least 10 mg/day prednisone equivalent for more than 2 weeks [11].

### Statistical analysis

Categorical data were summarized as frequencies presented as percentages. All tests were two-tailed. Statistical data were analyzed using SPSS Statistics (version 25; IBM, Armonk, NY, USA) unless otherwise indicated. Prism (version 7.04; GraphPad, San Diego, CA, USA) was used to calculate 95% CIs for sensitivity, specificity, PPV, and NPV. PLR and NLR were calculated using EZR (version 1.54; Saitama Medical Center, Jichi Medical University, Saitama, Japan).

## Results

### Characteristics of the cohorts

**Fig. 1:**
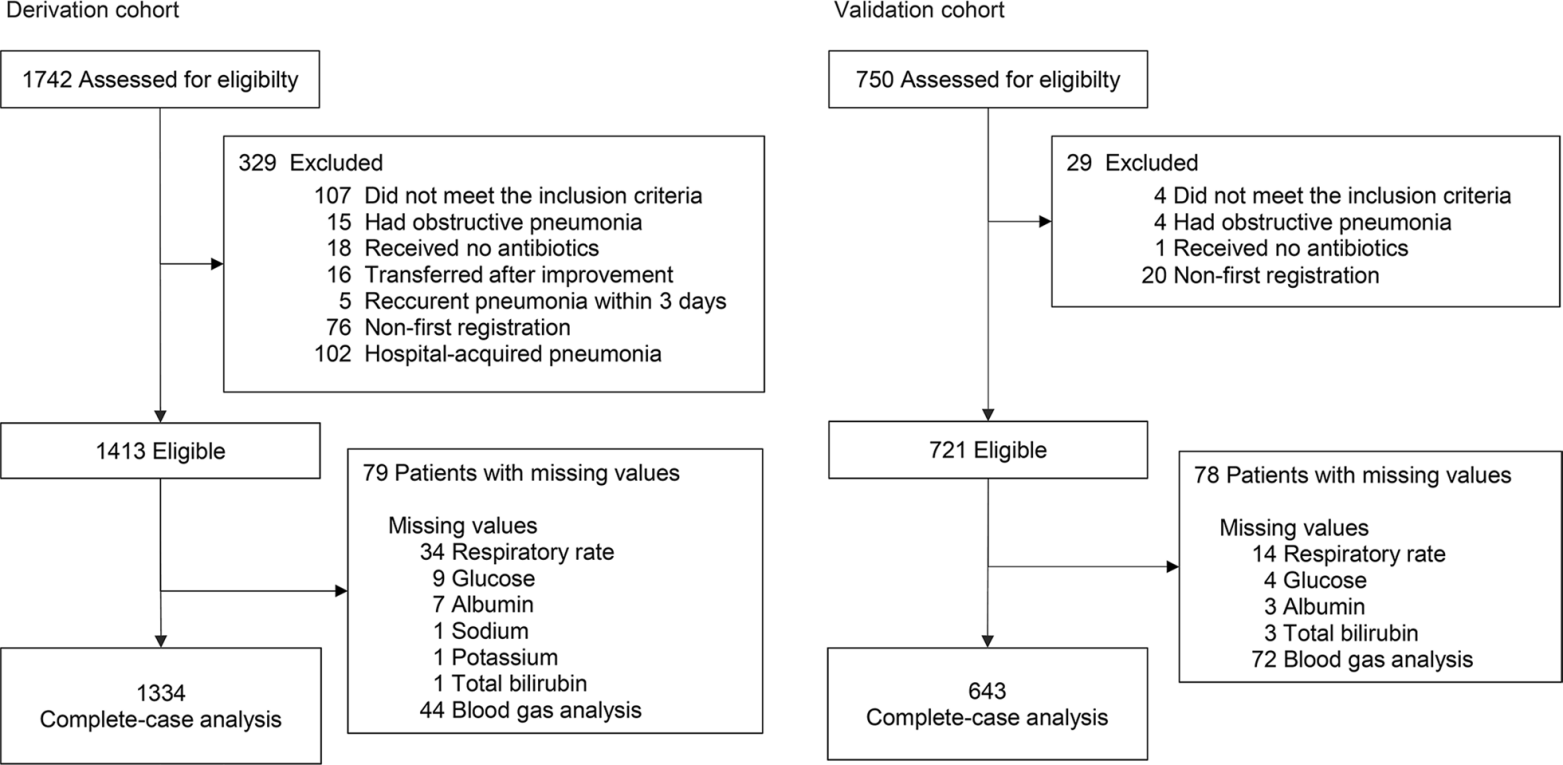
Patient flowchart in the derivation and validation cohorts. Model development and validation were performed in patients without missing values (complete-case analysis)

A total of 1413 patients were eligible in the derivation cohort, and 721 patients were included in the validation cohort. After excluding patients with missing data, the derivation and validation cohorts comprised 1334 and 643 patients, respectively (Fig. 1). The baseline patient characteristics were closely comparable between the derivation and validation cohorts (Table 1). The percentages of patients aged 80 years or older were identical in both the derivation and validation cohorts (42%). The percentages of patients with hyperpotassemia (K ≥ 5.0 mEq/L) and hypercapnia (PaCO_2_ > 45 mmHg) were higher in the derivation cohort than in the validation one. The incidence proportions of SAEs in the derivation and validation cohorts were 20.8% and 18.7%, respectively (Table 1).

### Prediction model development for SAEs in the derivation cohort

**Fig. 2:**
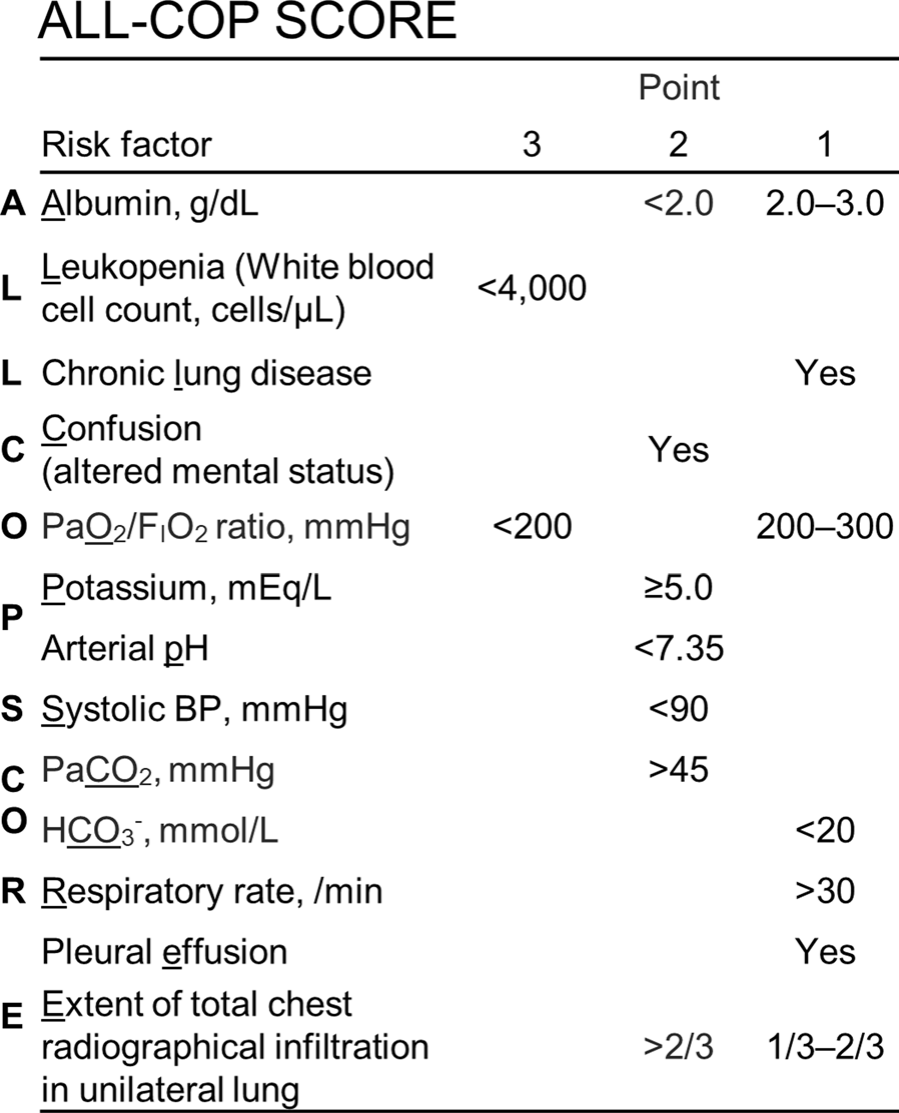
ALL-COP SCORE rule. The ALL-COP SCORE, a scoring system that was developed based on a multivariate logistic regression model, comprises 13 variables. Each variable is assigned between 1 and 3 points by rounding β-coefficients (Table 2). The point range of the ALL-COP SCORE is between 0 (min) and 24 (max)

**Fig. 3:**
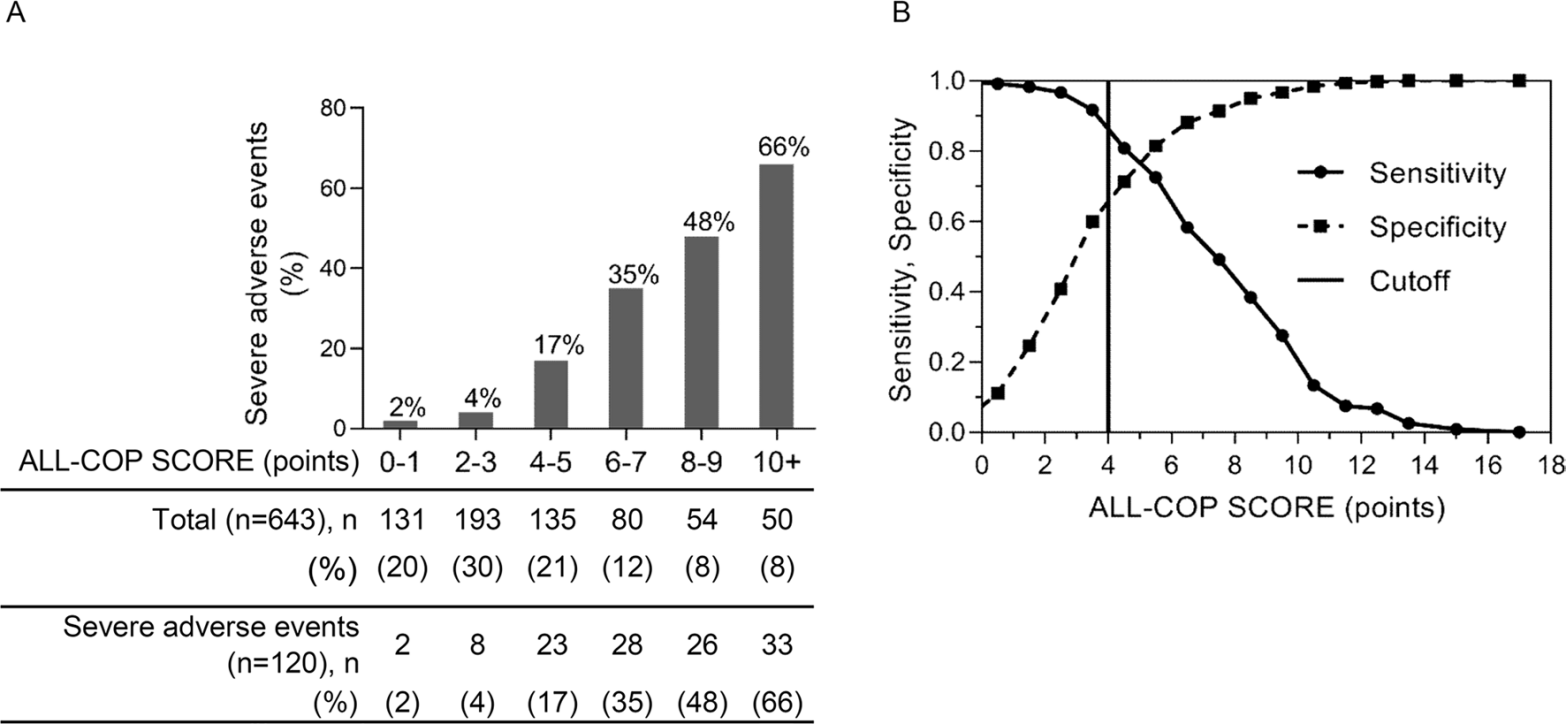
**A** Prevalence of patients with severe adverse events according to ALL-COP SCORE points in the validation cohort. **B** Sensitivity and specificity of the ALL-COP SCORE rule in the validation cohort. An ALL-COP SCORE ≥ 4 points has a sensitivity of 92% and a specificity of 60%

The results of the univariate analysis between 33 candidate variables and SAEs are shown in Additional file 2. As a result of the multivariate logistic regression analysis with forward stepwise selection, the prediction model for SAEs comprised 13 significant variables (Table 2). By rounding the β-coefficients of these variables, a simple scoring system was developed, named ALL-COP SCORE rule (Table 2, Fig. 2), which included the following variables: serum albumin level (< 2 g/dL, 2 points; 2–3 g/dL, 1 point); leukopenia (white blood cell count; < 4000 cells/μL, 3 points); chronic lung disease (1 point); confusion (2 points); PaO_2_/F_I_O_2_ ratio (< 200 mmHg, 3 points; 200–300 mmHg, 1 point); potassium level (≥ 5.0 mEq/L, 2 points); arterial pH (< 7.35, 2 points); systolic blood pressure (< 90 mmHg, 2 points); PaCO_2_ (> 45 mmHg, 2 points); HCO_3_^−^ (< 20 mmol/L, 1 point); respiratory rate (≥ 30 breaths/min, 1 point); pleural effusion (1 point); and extent of total chest radiographical infiltration of unilateral lung (> 2/3, 2 points; 1/2–2/3, 1 point). The ROC curve was similar between the ALL-COP SCORE rule and the original logistic regression model, and the AUROC was the same between the two models (0.84 [95% CI, 0.81–0.86] and 0.84 [95% CI, 0.81–0.87] for the ALL-COP SCORE rule and the original logistic regression model, respectively) (Additional file 3A). The sensitivity, specificity, and Youden’s index (sensitivity plus specificity minus one) for each point of the ALL-COP SCORE rule in the derivation cohort are shown in Additional file 3B. Since an ALL-COP SCORE threshold of ≥ 4 points had a specificity of ≥ 60% (Fig. 3, Additional file 3B) and as higher sensitivity was preferable to determine inpatients who should receive higher-intensity treatment, as described in the Methods, we determined ≥ 4 points as the ALL-COP SCORE threshold for identifying patients with CAP at high risk for SAEs at the time of pneumonia diagnosis.

**Table 2:** Multivariate analysis for severe adverse events in the derivation cohort

Variables	Severe adverse events*		Multivariate analysis		Points assigned^†^
	Yes (n = 277)	No (n = 1057)	β-coefficient	OR (95% CI)	
Intercept		− 3.82			
*Chronic lung diseases*					
No	166	717	0	1 (Ref)	
Yes	111	340	0.31	1.36 (0.97–1.93)	1
*Altered mental status*					
No	158	910	0	1 (Ref)	
Yes	119	147	0.76	2.14 (1.48–3.08)	2
*Systolic blood pressure, mmHg*					
≥ 90	247	1013	0	1 (Ref)	
< 90	30	44	0.61	1.85 (1.02–3.35)	2
*Respiratory rate, /min*					
< 30	169	854	0	1 (Ref)	
≥ 30	108	203	0.30	1.35 (0.95–1.92)	1
*Albumin, g/dL*					
≥ 3.0	134	749	0	1 (Ref)	
2.0–3.0	128	288	0.44	1.55 (1.11–2.19)	1
< 2.0	15	20	0.74	2.09 (0.90–4.86)	2
*Potassium, mEq/L*					
< 5.0	226	986	0	1 (Ref)	
≥ 5.0	51	71	0.80	2.22 (1.39–3.56)	2
*White blood cell count, cells/μL*					
≥ 4000	254	1036	0	1 (Ref)	
< 4000	23	21	1.24	3.45 (1.68–7.11)	3
*PaO*_*2*_*/F*_*I*_*O*_*2*_* ratio, mmHg*					
> 300	47	485	0	1 (Ref)	
200–300	85	397	0.52	1.68 (1.10–2.55)	1
100–200	82	127	1.02	2.76 (1.71–4.45)	3
≤ 100	63	48	1.40	4.07 (2.28–7.26)	3
*Arterial pH*					
≥ 7.35	193	993	0	1 (Ref)	
< 7.35	84	64	0.82	2.27 (1.41–3.66)	2
*PaCO*_*2*_*, mmHg*					
≤ 45	182	940	0	1 (Ref)	
> 45	95	117	0.91	2.47 (1.60–3.82)	2
*HCO*_*3*_^*−*^*, mmol/L*					
≥ 20	218	946	0	1 (Ref)	
< 20	59	111	0.59	1.80 (1.14–2.83)	1
*Pleural effusion*					
No	178	832	0	1 (Ref)	
Yes	99	225	0.52	1.68 (1.18–2.39)	1
*Extent of total infiltration*					
< 1/3 of unilateral lung	71	580	0	1 (Ref)	
1/3–2/3 of unilateral lung	100	315	0.48	1.62 (1.09–2.39)	1
> 2/3 of unilateral lung	106	162	0.74	2.10 (1.36–3.24)	2

Definition of abbreviations: OR, odds ratio; CI, confidence interval; Ref, reference

^*^Severe adverse events included death and requirement of mechanical ventilation (invasive or noninvasive) or vasopressor support within 30 days after pneumonia diagnosis

^†^Points were developed by rounding β-coefficients as follows: β-coefficient: 0.2–0.6, 1 point; 0.6–1.0, 2 points; and 1.0–1.4, 3 points

### Prediction model performance for SAEs in the validation cohort

**Fig. 4:**
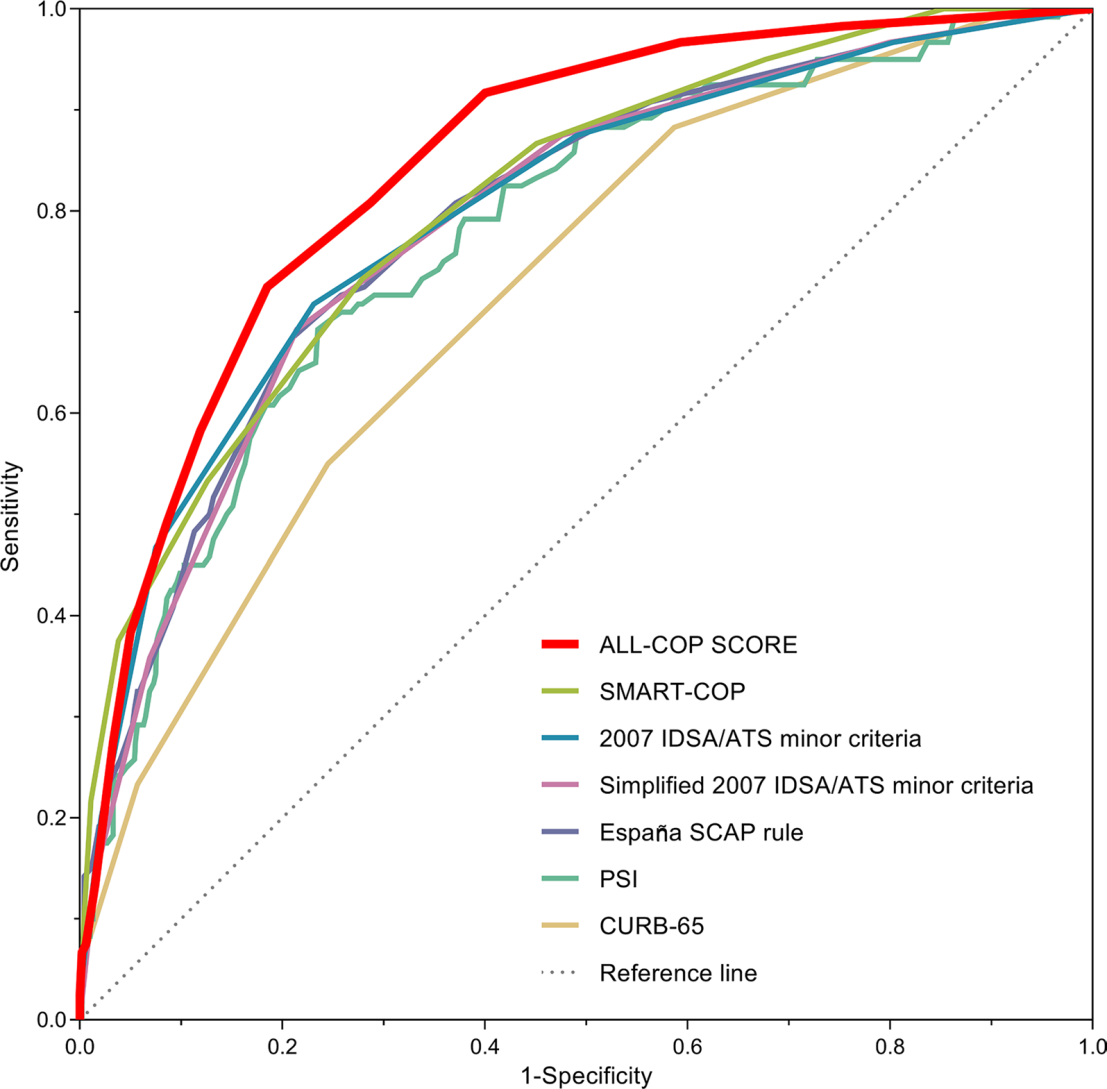
Comparison of the receiver operating characteristic curves of the prediction rules for severe adverse events in the validation cohort. Definition of abbreviations: SMART-COP, systolic blood pressure, multilobar chest X-ray involvement, albumin, respiratory rate, tachycardia, confusion, oxygenation, and arterial pH; IDSA/ATS, Infectious Disease Society of America/American Thoracic Society; SCAP, severe community-acquired pneumonia; PSI, Pneumonia Severity Index; CURB-65, confusion, urea level, respiratory rate, blood pressure, and age ≥ 65 years

Figure 3A shows the probabilities of SAEs within 30 days after pneumonia diagnosis according to the ALL-COP SCORE rule in the validation cohort. Patients with 4–5, 6–7, and ≥ 8 points had 17%, 35%, and 52% increase in the probability of SAEs, respectively, whereas the probability of SAEs was 3% in patients with ≤ 3 points. An ALL-COP SCORE threshold of ≥ 4 points had a sensitivity of 91.7% and a specificity of 60.0%, with a PPV of 34.5%, a NPV of 96.9%, a PLR of 2.3, and a NLR of 0.1. The ALL-COP SCORE rule exhibited a higher AUROC (0.85) compared with the other predictive models (Fig. 4, Table 3). The sensitivity of the ALL-COP SCORE rule was higher than those of the España SCAP rule, SMART-COP, 2007 IDSA/ATS minor criteria, and simplified 2007 IDSA/ATS minor criteria. Comparison of the sensitivities of the tested predictive models at the same level of specificity (0.60, the specificity of ≥ 4 points by the ALL-COP SCORE rule) and the same comparisons conducted after changing the thresholds so that the specificities were close to between 60 and 70% among the tested models revealed that the sensitivity of the ALL-COP SCORE rule was the highest among the evaluated predictive models (Fig. 4, Table 3).

**Table 3:** Comparison of the prediction rules for adverse outcomes in the validation cohort

Rule	Cutoff	Sensitivity, % (95% CI)	Specificity, % (95% CI)	PPV, % (95% CI)	NPV, % (95% CI)	PLR (95% CI)	NLR (95% CI)	AUROC (95% CI)
ALL-COP SCORE	≥ 4 Points	91.7 (85.3–95.4)	60.0 (55.8–64.2)	34.5 (29.5–39.9)	96.9 (94.4–98.3)	2.3 (2.0–2.6)	0.1 (0.1–0.3)	0.85 (0.81–0.88)
	≥ 5 Points	80.8 (72.9–86.9)	71.3 (67.3–75.0)	39.3 (33.4–45.5)	94.2 (91.4–96.1)	2.8 (2.4–3.3)	0.3 (0.2–0.4)	
	≥ 6 Points	72.5 (63.9–79.7)	81.5 (77.9–84.6)	47.3 (40.2–54.5)	92.8 (90.1–94.8)	3.9 (3.2–4.8)	0.3 (0.3–0.5)	
SMART-COP	≥ 3 Points (original)	86.7 (79.4–91.6)	54.9 (50.6–59.1)	30.6 (25.9–35.7)	94.7 (91.6–96.7)	1.9 (1.7–2.2)	0.2 (0.2–0.4)	0.81 (0.77–0.85)
	≥ 4 Points	73.3 (64.8–80.4)	72.1 (68.1–75.8)	37.6 (31.7–44.0)	92.2 (89.2–94.4)	2.6 (2.2–3.1)	0.4 (0.3–0.5)	
2007 IDSA/ATS criteria	Major criteria and/or ≥ 3 minor criteria (original)	72.5 (63.9–79.7)	76.9 (73.1–80.3)	41.8 (35.3–48.6)	92.4 (89.5–94.6)	3.1 (2.6–3.8)	0.4 (0.3–0.5)	–
	Major criteria and/or ≥ 2 minor criteria	87.5 (80.4–92.3)	50.9 (46.6–55.1)	29.0 (24.6–33.9)	94.7 (91.4–96.7)	1.8 (1.6–2.0)	0.2 (0.2–0.4)	
2007 IDSA/ATS minor criteria	≥ 3 Minor criteria (original)	70.8 (62.2–78.2)	76.9 (73.1–80.3)	41.3 (34.8–48.1)	92.0 (89.1–94.2)	3.1 (2.5–3.7)	0.4 (0.3–0.5)	0.80 (0.75–0.85)
	≥ 2 Minor criteria	87.5 (80.4–92.3)	50.9 (46.6–55.1)	29.0 (24.6–33.9)	94.7 (91.4–96.7)	1.8 (1.6–2.0)	0.2 (0.2–0.4)	
Simplified 2007 IDSA/ATS minor criteria	≥ 3 Minor criteria (original)	68.3 (59.6–76.0)	78.6 (74.9–81.9)	42.3 (35.5–49.3)	91.5 (88.6–93.8)	3.2 (2.6–3.9)	0.4 (0.3–0.5)	0.79 (0.74–0.83)
	≥ 2 Minor criteria	87.5 (80.4–92.3)	52.4 (48.1–56.6)	29.7 (25.1–34.6)	94.8 (91.6–96.8)	1.8 (1.6–2.1)	0.2 (0.1–0.4)	
España SCAP rule	≥ 10 Points (original)	88.3 (81.4–92.9)	49.0 (44.7–53.2)	28.4 (24.1–33.2)	94.8 (91.5–96.9)	1.7 (1.6–1.9)	0.2 (0.1–0.4)	0.79 (0.75–0.84)
	≥ 11 Points	80.8 (72.9–86.9)	62.9 (58.7–66.9)	33.3 (28.2–38.9)	93.5 (90.4–95.6)	2.2 (1.9–2.5)	0.3 (0.2–0.4)	
PSI	Classes IV and V (original)	92.5 (86.4–96.0)	35.6 (31.6–39.8)	24.8 (21.0–29.0)	95.4 (91.5–97.6)	1.4 (1.3–1.6)	0.2 (0.1–0.4)	0.78 (0.73–0.82)
	Class V	61.7 (52.7–69.9)	80.3 (76.7–83.5)	41.8 (34.8–49.2)	90.1 (87.1–92.5)	3.1 (2.5–3.9)	0.5 (0.4–0.6)	
CURB-65	≥ 3 Points (original)	55.0 (46.1–63.6)	75.5 (71.7–79.0)	34.0 (27.7–40.9)	88.0 (84.6–90.7)	2.2 (1.8–2.8)	0.6 (0.5–0.7)	0.72 (0.67–0.77)
	≥ 2 Points	88.3 (81.4–92.9)	41.3 (37.2–45.6)	25.7 (21.7–30.1)	93.9 (90.0–96.3)	1.5 (1.4–1.7)	0.3 (0.2–0.5)	

Definition of abbreviations: CI, confidence interval; PPV, positive predictive value; NPV, negative predictive value; PLR, positive likelihood ratio; NLR, negative likelihood ratio; AUROC, area under the receiver operating characteristic curve; SMART-COP, systolic blood pressure, multilobar chest X-ray involvement, albumin, respiratory rate, tachycardia, confusion, oxygenation, and arterial PH; IDSA/ATS, Infectious Disease Society of America/American Thoracic Society; SCAP, severe community-acquired pneumonia; PSI, Pneumonia Severity Index; CURB-65, confusion, urea level, respiratory rate, blood pressure, and age ≥ 65 years

### Subgroup analyses

Table 4 shows the results of the predictive performance of the ALL-COP SCORE rule in patient subgroups. The predictivity of the ALL-COP SCORE rule was more accurate when the rule was applied to patients without potential treatment restrictions. Specifically, compared to all patients in the validation cohort, the sensitivity was the same (91.2%), whereas the specificity increased by 10% (70.5%) (Table 3) in the subgroup of patients without potential treatment restrictions, which included 280 (81.9%) patients with CAP excluding HCAP and 62 (18.1%) patients with HCAP. The assessment of patients without invasive MV/VS at admission and those without immunosuppression revealed that the predictive performance of the ALL-COP SCORE rule was almost identical between each subgroup and the entire validation cohort.

**Table 4:** Performance of the ALL-COP SCORE for predicting severe adverse events in validation cohort subgroups

Subgroups	n	Severe adverse events*, n (%)	Sensitivity, % (95% CI)	Specificity, % (95% CI)	PPV, % (95% CI)	NPV, % (95% CI)	PLR (95% CI)	NLR (95% CI)	AUROC (95% CI)
Patients without potential treatment restriction^†^	342	57 (16.7)	91.2 (81.1–96.2)	70.5 (65.0–75.5)	38.2 (30.5–46.6)	97.6 (94.4–99.0)	3.1 (2.5–3.8)	0.1 (0.1–0.3)	0.89 (0.84–0.94)
CAP excluding HCAP^‡^	437	71 (16.2)	94.4 (86.4–97.8)	65.0 (60.0–69.7)	34.3 (28.1–41.3)	98.4 (95.8–99.4)	2.7 (2.3–3.1)	0.1 (0.0–0.2)	0.88 (0.84–0.92)
HCAP^§^	206	49 (23.8)	87.8 (75.8–94.3)	48.4 (40.7–56.2)	34.7 (26.9–43.4)	92.7 (84.9–96.6)	1.7 (1.4–2.0)	0.3 (0.1–0.5)	0.78 (0.70–0.85)
Patients without requirement of invasive MV/VS at admission^||^	629	106 (16.9)	91.5 (84.7–95.5)	60.0 (55.8–64.2)	31.7 (26.7–37.1)	97.2 (95.0–98.5)	2.3 (2.0–2.6)	0.1 (0.1–0.3)	0.84 (0.80–0.88)
Patients without immunosuppression**	585	105 (17.9)	91.4 (84.5–95.4)	60.0 (56.0–64.7)	33.6 (28.3–39.2)	97.0 (94.4–98.4)	2.3 (2.0–2.6)	0.1 (0.1–0.3)	0.84 (0.80–0.88)

Definition of abbreviations: CI, confidence interval; PPV, positive predictive value; NPV, negative predictive value; PLR, positive likelihood ratio; NLR, negative likelihood ratio; AUROC, area under the receiver operating characteristic curve; CAP, community-acquired pneumonia; HCAP, healthcare-associated pneumonia; MV, mechanical ventilation; VS, vasopressor support

^*^Severe adverse events included death and requirement of mechanical ventilation (invasive or noninvasive) or vasopressor support within 30 days after pneumonia diagnosis

^†^Patients without potential treatment restriction were defined as those under 80 years and without nonambulatory status (who were able to walk)

^‡^CAP excluding HCAP was defined as pneumonia that did not match the criteria for hospital-acquired pneumonia (pneumonia occurring 48 h or more after hospital admission)

^§^HCAP was defined as pneumonia co-occurring with any of the following conditions: hospitalization for 2 days or more during the preceding 90 days, residence in a nursing home or extended care facility, home intravenous therapy (including antibiotics and chemotherapy), chronic dialysis (including hemodialysis and peritoneal dialysis) during the preceding 30 days, or home wound care during the preceding 30 days

^||^Patients who required invasive MV/VS at admission were excluded

^**^Immunosuppression included any immunosuppressive diseases, such as congenital or acquired immunodeficiency, hematologic diseases, asplenia and neutropenia (< 1000 cells/μL), treatment with immunosuppressive agents, chemotherapy within the previous 30 days, or corticosteroids in daily doses of at least 10 mg/day of a prednisone equivalent for more than 2 weeks

The assessment for the performance of the ALL-COP SCORE rule in secondary endpoints in the validation cohort revealed that an ALL-COP SCORE threshold of ≥ 4 points had a sensitivity of 90.0% and a specificity of 54.6% for 30-day mortality, a sensitivity of 93.2% and a specificity of 56.1% for the requirement of MV/VS, and a sensitivity of 90.9% and a specificity of 55.1% for ICU admission (Additional file 4).

## Discussion

In the present study, we developed and validated the ALL-COP SCORE prediction rule for its utility in all patients with CAP including those with HCAP. The ALL-COP SCORE rule exhibited a high AUROC (0.85), and an ALL-COP SCORE threshold of ≥ 4 points showed a high sensitivity (92%) with 60% specificity for predicting SAEs. These results indicated that the proposed ALL-COP SCORE prediction rule may be useful for identifying patients at high risk for SAEs at the time of CAP diagnosis.

The optimal definition of severe CAP, which remains a topic of debate [5–9, 23], should be used as a guide during decision-making on identifying patients who require a higher level of inpatient treatment intensity. The 30-day mortality was adopted as a primary outcome measure to identify patients with mild CAP who could be treated as outpatients, as described in the original studies on the PSI and CURB-65 [1–3, 24, 25]. Conversely, several previous studies adopted ICU admission as the primary outcome measure to identify patients who should receive higher-intensity treatment for severe pneumonia [26–29]. However, some concerns remain that using ICU admission as an outcome measure might prevent the generalizability of findings due to regional differences in the ICU admission criteria depending on medical resources [5, 6, 9, 21]. Thus, España and colleagues adopted a composite endpoint including MV, septic shock, and death to develop the España SCAP rule [5], whereas Charles and colleagues employed intensive respiratory or VS as a primary outcome measure to develop the SMART-COP [6]. Regarding this outcome measure, some researchers adovocate death should not be combined with MV or VS. However, Torsten and colleagues showed that, even patients residing in nursing homes and bedridden patients were excluded, 76.3% of nonsurvivors of CAP did not receive ventilatory support (62.6% of those aged < 65 years) [30]. They stated that the number of nonsurvivors without obvious reasons for withholding ventilatory support was high, particularly in younger patients, and suggested that these high percentages might reflect either treatment restrictions or deficient clinical performance. Therefore, considering future contribution to improve the clinical performance, we defined SAEs as a primary outcome measure and included death and requirement of MV or VS within 30 days after pneumonia diagnosis.

We considered that a model with high sensitivity would be preferable when identifying patients at high risk for SAEs and those who should receive higher-intensity treatment. The 2019 ATS/IDSA CAP guidelines suggest that the 2007 IDSA/ATS severity criteria should be used. However, the AUROC of the ALL-COP SCORE rule was highest among the tested predictive models including the 2007 IDSA/ATS criteria. The sensitivity of the ALL-COP SCORE rule was high at 92%, with a specificity of 60%, when the threshold to identify patients with SAEs was set at ≥ 4 points. The sensitivity of the ALL-COP SCORE rule was the highest when compared to the other tested predictive rules at the same specificity level of 60%. Several reasons might potentially explain the high predictive ability of the ALL-COP SCORE rule. First, the ALL-COP SCORE rule includes several variables such as hypercapnia and hyperkalemia, which are not included in the other tested prediction models, although they are reported as prognostic factors for pneumonia [16, 17]. Second, we adopted ternary or quaternary variables, as well as binary variables, because ternary or quaternary variables provided more accurate effects for SAEs compared with the binary variables that were used in the existing prediction rules [1–3, 5, 6]. Findings of the present study also suggest that the examination of some variables, such as serum albumin, PaO_2_, and pH, which might not be universally available for real-time clinical decision-making [4], might still be worthwhile to assess at the time of pneumonia diagnosis to improve patient care.

The predictive performance of the ALL-COP SCORE rule was improved when patients with possible treatment restrictions were excluded, suggesting that this prediction model may be more useful as a decision-making tool to identify patients requiring intensive care among those without treatment restrictions. Specifically, in the absence of treatment restrictions, physicians should aggressively consider higher-intensity treatment at the time of pneumonia diagnosis in patients with ≥ 4 points according to the ALL-COP SCORE rule. Furthermore, these patients might be strongly recommended for early interventions such as CAP bundle of care [31] (e.g., rapid empirical antibiotic administration with a β-lactam and macrolide [32–34], rapid resuscitation, thromboembolic prophylaxis, and appropriate management for hypoxia [35–38]).

The present study has several limitations. First, the biggest one is that data in two cohorts used in this study was obtained before the coronavirus disease 2019 (COVID-19) pandemic. We are going to validate whether the ALL-COP SCORE rule can be useful in both patient groups of COVID-19 and non-COVID-19 pneumonia. One important future research question is whether prediction models for worse outcomes can be adopted in all CAP including COVID-19 or should be separately considered in COVID-19 and non-COVID-19. Second, there were 13 constitutive variables as part of the ALL-COP SCORE rule, more than those of the other predictive rules [1, 3, 5, 6, 20]. This limitation can be overcome if the ALL-COP SCORE rule is used as a calculation tool in a medical application software or can be combined with an electric medical record system. Third, the present study did not confirm do-not-resuscitate orders at the time of admission. Elderly patients or those with poor functional status might have had various potential treatment restrictions even in the absence of a do-not-resuscitate order. Thus, we defined patients aged 80 years or older and any patient without independent ambulation as those with potential treatment restrictions. Fourth, the present study was performed in Japan which might hinder the international generalizability of the ALL-COP SCORE rule. Therefore, international collaborative studies for further validation of this model are necessary in the future. Furthermore, the efficacy and safety of the ALL-COP SCORE rule as a decision aid to guide treatment intensity should be assessed.

## Conclusions

The ALL-COP SCORE is a potentially useful tool to predict patients at high risk for SAEs within 30 days after pneumonia diagnosis and can be adopted to all patients with CAP including those with HCAP. Higher-intensity treatment should be considered in patients with CAP and an ALL-COP SCORE threshold of ≥ 4 points.

## Supplementary Information

**Additional file 1.** Methodological Details. Details on pneumonia definitions and categories, inclusion and exclusion criteria, definitions of variables, procedures, and data collection.

**Additional file 2. Supplemental Table 1.** Univariate analysis for severe adverse events in the derivation cohort.

**Additional file 3. Supplemental Figure A, B.** Receiver operating characteristic curves of the multivariate logistic regression model and the ALL-COP SCORE rule in the derivation cohort (**A**). Sensitivity, specificity, and Youden's index at each point of the ALL-COP SCORE rule in the derivation cohort (**B**).

**Additional file 4. Supplemental Table 2.** Comparison of rules predicting for secondary endpoints in the validation cohort.

## Data Availability

Original data and analyses are available from the corresponding author on reasonable request.

## Abbreviations

CAP: Community-acquired pneumonia
PSI: Pneumonia Severity Index
CURB-65: Confusion, blood urea nitrogen > 7 mmol/L [20 mg/dL], respiratory rate ≥ 30/min, low blood pressure [diastolic blood pressure ≤ 60 mm Hg or systolic blood pressure < 90 mm Hg], and age ≥ 65 years
SAE: Severe adverse event
IDSA: Infectious Diseases Society of America
ATS: American Thoracic Society
HCAP: Healthcare-associated pneumonia
MV: Mechanical ventilation
VS: Vasopressor support
ICU: Intensive care unit
OR: Odds ratio
CI: Confidence interval
ROC: Receiver operating characteristic
PPV: Positive predictive value
NPV: Negative predictive value
AUROC: Area under the receiver operating characteristic curve
COVID-19: Coronavirus disease 2019
