# Supplementary material for: A prediction rule for severe adverse events in all inpatients with community-acquired pneumonia: a multicenter observational study
Source: BMC Pulm Med. 2022 Jan 12;22:34. doi: 10.1186/s12890-022-01819-0 (PMC8753951; doi:10.1186/s12890-022-01819-0)
Supplement: Supplementary file 1 — Additional file 1. Methodological Details. Details on pneumonia definitions and categories, inclusion and exclusion criteria, definitions of variables, procedures, and data collection. [file 12890_2022_1819_MOESM1_ESM.docx]

Additional file 1

**Methodological Details**

**Definition of Pneumonia and Exclusion Criteria**

Pneumonia was defined as the presence of a new infiltrate on a chest radiograph in addition to at least two of the following: new or increased cough or sputum production, new pleuritic chest pain, new or increased dyspnea, fever (temperature ≥ 38.0°C) or hypothermia (temperature < 35.0°C), leukocytosis [white blood cell (WBC) count ≥ 10,000 cells/μL] or leukopenia (WBC count < 4,000 cells/μL), or new hypoxemia [blood oxygen saturation level (SpO_2_) < 90% or arterial blood oxygen tension (PaO_2_) < 60 mmHg], or deterioration of hypoxemia [[1](#_ENREF_1), [2](#_ENREF_2)]. Exclusion criteria were as follows: (1) patients with obstructive pneumonia, (2) patients whose pneumonia recurred within three days after the last administration of antibiotics, (3) patients who did not receive initial antibiotic treatment, (4) patients who were transferred from other institutions and were already clinically improved, and (5) patients who had been previously enrolled in this study [[3](#_ENREF_3), [4](#_ENREF_4)].

**Categories of Pneumonia**

Community-acquired pneumonia was defined as pneumonia which occurred outside the hospital or within 48 hours after hospital admission. Community-acquired pneumonia included healthcare-associated pneumonia (HCAP). HCAP was defined as pneumonia co-occurring with hospitalization for 2 days or more during the preceding 90 days, residence in a nursing home or extended-care facility, home intravenous therapy (including antibiotics and chemotherapy), chronic dialysis (including hemodialysis and peritoneal dialysis) during the preceding 30 days, or home wound care during the preceding 30 days [[1](#_ENREF_1)]. Hospital-acquired pneumonia (HAP) was defined as pneumonia occurring 48 hours more after hospital admission, including ventilator-associated pneumonia [[1](#_ENREF_1)].

**Definitions of Comorbidities and Patient Status**

Chronic pulmonary disease included simple chronic bronchitis, chronic obstructive pulmonary disease, and structural lung diseases such as bronchiectasis and interstitial lung disorders. Immunosuppression included any immunosuppressive diseases, such as congenital or acquired immunodeficiency, hematologic diseases, asplenia, and neutropenia (< 1,000 cells/μL); treatment with immunosuppressive drugs within the previous 30 days; administration of corticosteroids at daily dose equivalent to ≥ 10 mg/day of prednisone for more than 2 weeks; or administration of chemotherapy within the previous 30 days [[3](#_ENREF_3)]. Nonambulatory status was defined as being bedridden or using a wheelchair because of walking difficulty

**References**

1. American Thoracic Society, Infectious Diseases Society of America: **Guidelines for the management of adults with hospital-acquired, ventilator-associated, and healthcare-associated pneumonia**. *Am J Respir Crit Care Med* 2005, **171**(4):388-416.

2. Mandell LA, Wunderink RG, Anzueto A, Bartlett JG, Campbell GD, Dean NC, Dowell SF, File TM, Jr., Musher DM, Niederman MS *et al*: **Infectious Diseases Society of America/American Thoracic Society consensus guidelines on the management of community-acquired pneumonia in adults**. *Clin Infect Dis* 2007, **44 Suppl 2**:S27-72.

3. Shindo Y, Ito R, Kobayashi D, Ando M, Ichikawa M, Shiraki A, Goto Y, Fukui Y, Iwaki M, Okumura J *et al*: **Risk Factors for Drug-Resistant Pathogens in Community-Acquired and Healthcare-Associated Pneumonia**. *Am J Respir Crit Care Med* 2013, **188**(8):985-995.

4. Kobayashi D, Shindo Y, Ito R, Iwaki M, Okumura J, Sakakibara T, Yamaguchi I, Yagi T, Ogasawara T, Sugino Y *et al*: **Validation of the prediction rules identifying drug-resistant pathogens in community-onset pneumonia**. *Infect Drug Resist* 2018, **11**:1703-1713.
