# Supplementary material for: A prediction rule for severe adverse events in all inpatients with community-acquired pneumonia: a multicenter observational study
Source: BMC Pulm Med. 2022 Jan 12;22:34. doi: 10.1186/s12890-022-01819-0 (PMC8753951; doi:10.1186/s12890-022-01819-0)
Supplement: Supplementary file 2 — Additional file 2. Supplemental Table 1. Univariate analysis for severe adverse events in the derivation cohort. [file 12890_2022_1819_MOESM2_ESM.docx]

Additional file 2

**Univariate analysis for severe adverse events in the derivation cohort.**

| Variables | | Severe adverse eventes^*^ | | Univariate analysis | |
| --- | --- | --- | --- | --- | --- |
|  |  | Yes  (n=277) | No  (n=1057) | OR (95% CI) | p-value |
| Age, yr | |  |  |  |  |
|  | < 65 | 42 | 208 | 1 (Ref) |  |
|  | 65–79 | 105 | 423 | 1.23 (0.83–1.82) | 0.305 |
|  | ≥ 80 | 130 | 426 | 1.51 (1.03–2.22) | 0.036 |
| Gender | |  |  |  |  |
|  | Female | 88 | 375 | 1 (Ref) |  |
|  | Male | 189 | 682 | 1.18 (0.89–1.57) | 0.249 |
| Nursing home resident | |  |  |  |  |
|  | No | 214 | 904 | 1 (Ref) |  |
|  | Yes | 63 | 153 | 1.74 (1.25–2.42) | 0.001 |
| Nonamburatory status | |  |  |  |  |
|  | No | 175 | 832 | 1 (Ref) |  |
|  | Yes | 102 | 225 | 2.16 (1.62–2.87) | <0.001 |
| Heavy alcohol use | |  |  |  |  |
|  | No | 265 | 1029 | 1 (Ref) |  |
|  | Yes | 12 | 28 | 1.66 (0.84–3.32) | 0.148 |
| Neoplastic diseases | |  |  |  |  |
|  | No | 233 | 910 | 1 (Ref) |  |
|  | Yes | 44 | 147 | 1.17 (0.81–1.69) | 0.403 |
| Congestive heart failure | |  |  |  |  |
|  | No | 222 | 935 | 1 (Ref) |  |
|  | Yes | 55 | 122 | 1.90 (1.34–2.70) | <0.001 |
| Chronic liver diseases | |  |  |  |  |
|  | No | 263 | 1020 | 1 (Ref) |  |
|  | Yes | 14 | 37 | 1.47 (0.78–2.76) | 0.233 |
| Cerebrovascular diseases | |  |  |  |  |
|  | No | 208 | 835 | 1 (Ref) |  |
|  | Yes | 69 | 222 | 1.25 (0.92–1.70) | 0.162 |
| Diabetes | |  |  |  |  |
|  | No | 224 | 859 | 1 (Ref) |  |
|  | Yes | 53 | 198 | 1.03 (0.73–1.44) | 0.879 |
| Immunosuppression^‡^ | |  |  |  |  |
|  | No | 249 | 967 | 1 (Ref) |  |
|  | Yes | 28 | 90 | 1.21 (0.77–1.89) | 0.406 |
| Chronic renal diseases | |  |  |  |  |
|  | No | 249 | 977 | 1 (Ref) |  |
|  | Yes | 28 | 80 | 1.37 (0.87–2.16) | 0.169 |
| Chronic lung diseases | |  |  |  |  |
|  | No | 166 | 717 | 1 (Ref) |  |
|  | Yes | 111 | 340 | 1.41 (1.07–1.85) | 0.014 |
| Altered mental status | |  |  |  |  |
|  | No | 158 | 910 | 1 (Ref) |  |
|  | Yes | 119 | 147 | 4.66 (3.47–6.26) | <0.001 |
| Pulse rate, /min | |  |  |  |  |
|  | < 125 | 230 | 969 | 1 (Ref) |  |
|  | ≥ 125 | 47 | 88 | 2.25 (1.54–3.30) | <0.001 |
| Body temperature, °C | |  |  |  |  |
|  | ≥ 36.0 | 254 | 1038 | 1 (Ref) |  |
|  | < 36.0 | 23 | 19 | 4.95 (2.65–9.22) | <0.001 |
| Systolic blood pressure, mmHg | |  |  |  |  |
|  | ≥ 90 | 247 | 1013 | 1 (Ref) |  |
|  | < 90 | 30 | 44 | 2.80 (1.72–4.54) | <0.001 |
| Respiratory rate, /min | |  |  |  |  |
|  | < 30 | 169 | 854 | 1 (Ref) |  |
|  | ≥ 30 | 108 | 203 | 2.69 (2.02–3.58) | <0.001 |
| Blood urea nitrogen, mg/dL | |  |  |  |  |
|  | < 30 | 174 | 862 | 1 (Ref) |  |
|  | ≥ 30 | 103 | 195 | 2.62 (1.96–3.49) | <0.001 |
| Glucose, mg/dL | |  |  |  |  |
|  | 70–249 | 238 | 980 | 1 (Ref) |  |
|  | < 70 | 13 | 6 | 8.92 (3.36–23.72) | <0.001 |
|  | ≥ 250 | 26 | 71 | 1.51 (0.94–2.42) | 0.087 |
| Albumin, g/dL | |  |  |  |  |
|  | ≥ 3.0 | 134 | 749 | 1 (Ref) |  |
|  | 2.0–3.0 | 128 | 288 | 2.48 (1.88–3.28) | <0.001 |
|  | < 2.0 | 15 | 20 | 4.19 (2.09–8.39) | <0.001 |
| Sodium, mEq/L | |  |  |  |  |
|  | 130–145 | 229 | 965 | 1 (Ref) |  |
|  | < 130 | 27 | 64 | 1.78 (1.11–2.85) | 0.017 |
|  | ≥ 146 | 21 | 28 | 3.16 (1.76–5.67) | <0.001 |
| Potassium, mEq/L | |  |  |  |  |
|  | < 5.0 | 226 | 986 | 1 (Ref) |  |
|  | ≥ 5.0 | 51 | 71 | 3.13 (2.13–4.62) | <0.001 |
| Total bilirubin, mg/dL | |  |  |  |  |
|  | < 2.0 | 266 | 1009 | 1 (Ref) |  |
|  | ≥ 2.0 | 11 | 48 | 0.87 (0.45–1.70) | 0.681 |
| White blood cell count, cells/μL | |  |  |  |  |
|  | ≥ 4000 | 254 | 1036 | 1 (Ref) |  |
|  | < 4000 | 23 | 21 | 4.47 (2.43–8.20) | <0.001 |
| Hematocrit, % | |  |  |  |  |
|  | ≥ 30.0 | 222 | 920 | 1 (Ref) |  |
|  | < 30.0 | 55 | 137 | 1.66 (1.18–2.35) | 0.004 |
| Platelet count, cells/μL | |  |  |  |  |
|  | ≥ 10×10^4^ | 260 | 1016 | 1 (Ref) |  |
|  | < 10×10^4^ | 17 | 41 | 1.62 (0.91–2.90) | 0.104 |
| PaO_2_/F_I_O_2_ ratio, mmHg | |  |  |  |  |
|  | > 300 | 47 | 485 | 1 (Ref) |  |
|  | 200–300 | 85 | 397 | 2.21 (1.51–3.23) | <0.001 |
|  | 100–200 | 82 | 127 | 6.66 (4.43–10.02) | <0.001 |
|  | ≤ 100 | 63 | 48 | 13.54 (8.38–21.89) | <0.001 |
| Arterial pH | |  |  |  |  |
|  | ≥ 7.350 | 193 | 993 | 1 (Ref) |  |
|  | < 7.350 | 84 | 64 | 6.75 (4.71–9.68) | <0.001 |
| PaCO_2_, mmHg | |  |  |  |  |
|  | ≤ 45.0 | 182 | 940 | 1 (Ref) |  |
|  | > 45.0 | 95 | 117 | 4.19 (3.06–5.74) | <0.001 |
| HCO_3_^-^, mmol/L | |  |  |  |  |
|  | ≥ 20.0 | 218 | 946 | 1 (Ref) |  |
|  | < 20.0 | 59 | 111 | 2.31 (1.63–3.27) | <0.001 |
| Pleural effusion | |  |  |  |  |
|  | No | 178 | 832 | 1 (Ref) |  |
|  | Yes | 99 | 225 | 2.06 (1.54–2.74) | <0.001 |
| Extent of total chest radiographical infiltration | | | |  |  |
|  | < 1/3 of unilateral lung | 71 | 580 | 1 (Ref) |  |
|  | 1/3–2/3 of unilateral lung | 100 | 315 | 2.59 (1.86–3.62) | <0.001 |
|  | > 2/3 of unilateral lung | 106 | 162 | 5.35 (3.78–7.57) | <0.001 |

Definition of abbreviations: OR = odds ratio; CI = confidence interval; Ref = reference.

^*^Severe adverse events included death and requirement of mechanical ventilation (invasive or non-invasive) or vasopressor support within 30 days after diagnosis of pneumonia.

^†^Immunosuppression included any immunosuppressive diseases, such as congenital or acquired immunodeficiency, hematologic diseases, asplenia and neutropenia (< 1,000 cells/μL), treatment with immunosuppressive agents, chemotherapy within the previous 30 days, or corticosteroids in daily doses of at least 10 mg/day of a prednisone equivalent for more than 2 weeks.
