# Supplementary material for: A prediction rule for severe adverse events in all inpatients with community-acquired pneumonia: a multicenter observational study
Source: BMC Pulm Med. 2022 Jan 12;22:34. doi: 10.1186/s12890-022-01819-0 (PMC8753951; doi:10.1186/s12890-022-01819-0)
Supplement: Supplementary file 3 — Additional file 3. Supplemental Figure A, B. Receiver operating characteristic curves of the multivariate logistic regression model and the ALL-COP SCORE rule in the derivation cohort (A). Sensitivity, specificity, and Youden's index at each point of the ALL-COP SCORE rule in the derivation cohort (B). [file 12890_2022_1819_MOESM3_ESM.docx]

Additional file 3


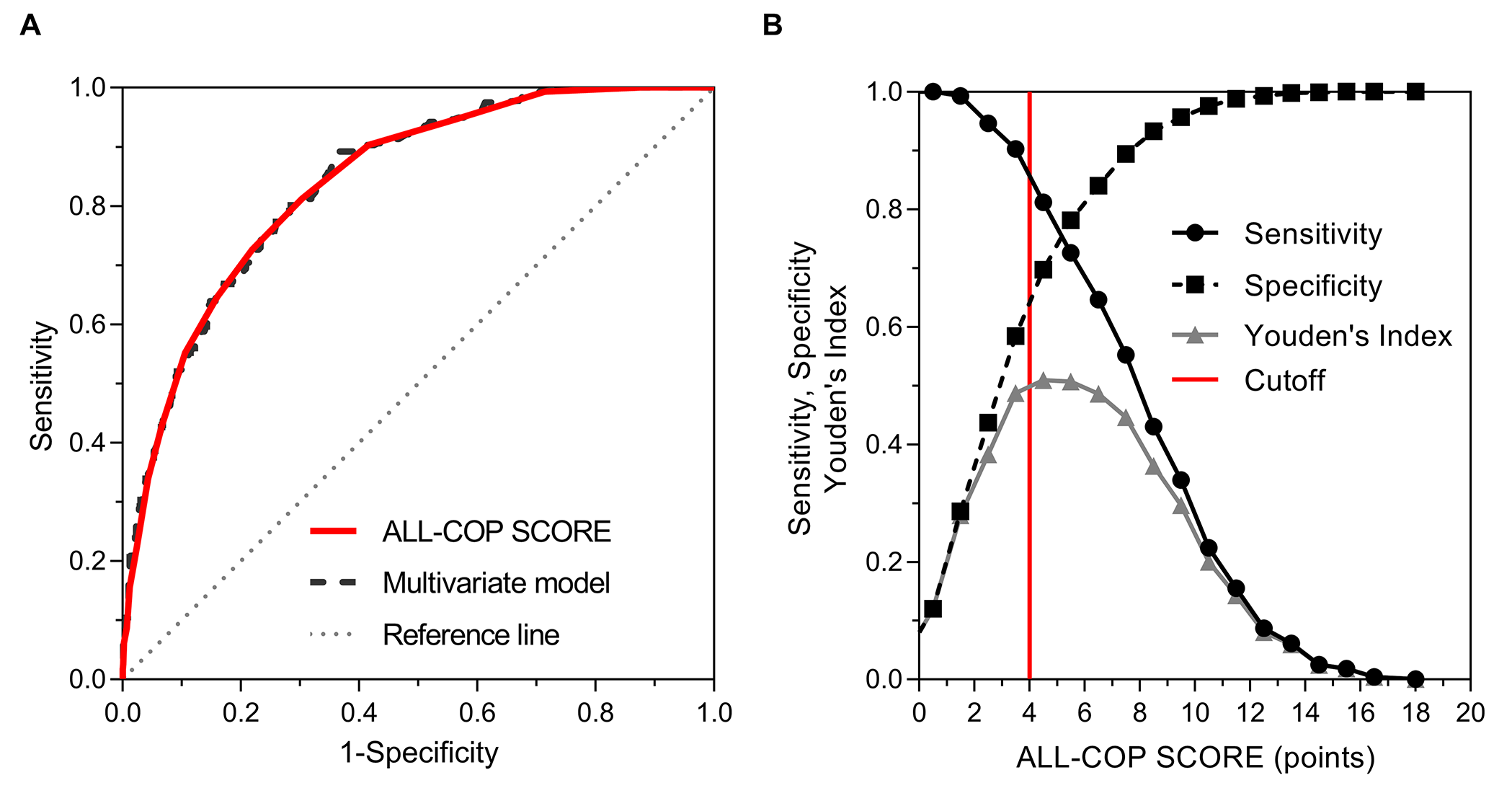


**A.** Receiver operating characteristic curves of the multivariate logistic regression model and the ALL-COP SCORE rule (simple scoring system developed from the multivariate logistic regression model) in the derivation cohort. The area under the receiver operating characteristics (AUROC) of the multivariate logistic regression model showed 0.84 (95% Confidence Interval [CI], 0.81–0.87), the AUROC of the ALL-COP SCORE rule showed 0.84 (95% CI, 0.81–0.86).

**B.** Sensitivity, specificity, and Youden's index (sensitivity plus specificity minus one) at each point of the ALL-COP SCORE rule in the derivation cohort. Youden’s index was high when cutoff was 4, 5, 6, or 7. Considering that higher sensitivity is preferable, we determined 4 or more points of the ALL-COP SCORE rule as a threshold for identifying patients with community-acquired pneumonia at high-risk of severe adverse events at the time point of pneumonia diagnosis.
