# Supplementary material for: A prediction rule for severe adverse events in all inpatients with community-acquired pneumonia: a multicenter observational study
Source: BMC Pulm Med. 2022 Jan 12;22:34. doi: 10.1186/s12890-022-01819-0 (PMC8753951; doi:10.1186/s12890-022-01819-0)
Supplement: Supplementary file 4 — Additional file 4. Supplemental Table 2. Comparison of rules predicting for secondary endpoints in the validation cohort. [file 12890_2022_1819_MOESM4_ESM.docx]

Additional file 4

**Comparison of rules predicting for secondary endpoints in the validation cohort**

| A. For 30-day mortality | | | | | | |  |
| --- | --- | --- | --- | --- | --- | --- | --- |
|  | Rule | Sensitivity,  % (95% CI) | Specificity,  % (95% CI) | PPV,  % (95% CI) | NPV,  % (95% CI) | AUROC  (95% CI) | |
|  | ALL-COP SCORE (≥ 4 points) | 90.0 (79.9–95.3) | 54.6 (50.5–58.5) | 16.9 (13.2–21.4) | 98.2 (96.0–99.2) | 0.79 (0.73–0.84) | |
|  | SMART-COP (≥ 3 points) | 81.7 (70.1–89.4) | 50.1 (46.0–54.1) | 14.4 (11.1–18.5) | 96.4 (93.6–98.0) | 0.74 (0.67–0.80) | |
|  | 2007 IDSA/ATS criteria | 68.3 (55.8–78.7) | 71.4 (67.6–74.9) | 19.7 (14.9–25.7) | 95.6 (93.3–97.2) | 0.72 (0.66–0.79) | |
|  | 2007 IDSA/ATS minor criteria (≥ 3 factors) | 68.3 (55.8–78.7) | 71.7 (67.9–75.2) | 19.9 (15.0–25.9) | 95.7 (93.3–97.2) | - | |
|  | Simplified 2007 IDSA/ATS minor criteria (≥ 3 factors) | 66.7 (54.1–77.3) | 73.6 (69.9–77.0) | 20.6 (15.5–26.9) | 95.6 (93.2–97.1) | 0.76 (0.69–0.82) | |
|  | España SCAP rule (≥ 10 points) | 86.7 (75.8–93.1) | 44.9 (41.0–49.0) | 13.9 (10.8–17.8) | 97.0 (94.3–98.5) | 0.74 (0.67–0.81) | |
|  | PSI (Classes IV and V) | 95.0 (86.3–98.6) | 32.9 (29.2–36.9) | 12.7 (10.0–16.1) | 98.5 (95.6–99.6) | 0.78 (0.72–0.84) | |
|  | CURB-65 (≥ 3 points) | 55.0 (42.5–66.9) | 72.4 (68.6–75.9) | 17.0 (12.4–22.9) | 94.0 (91.4–95.8) | 0.69 (0.62–0.75) | |
|  |  |  |  |  |  |  | |
| B. For requirement of mechanical ventilation or vasopressor support | | | | | | |  |
|  | Rule | Sensitivity,  % (95% CI) | Specificity,  % (95% CI) | PPV,  % (95% CI) | NPV,  % (95% CI) | AUROC  (95% CI) | |
|  | ALL-COP SCORE (≥ 4 points) | 93.2 (85.1–97.1) | 56.1 (52.0–60.1) | 21.6 (17.5–26.5) | 98.5 (96.4–99.3) | 0.84 (0.80–0.89) | |
|  | SMART-COP (≥ 3 points) | 91.9 (83.4–96.2) | 52.2 (48.1–56.3) | 20.0 (16.1–24.6) | 98.0 (95.8–99.1) | 0.79 (0.73–0.84) | |
|  | 2007 IDSA/ATS criteria | 74.3 (63.4–82.9) | 73.1 (69.3–76.6) | 26.4 (20.9–32.8) | 95.6 (93.3–97.2) | 0.83 (0.78–0.87) | |
|  | 2007 IDSA/ATS minor criteria (≥ 3 factors) | 71.6 (60.5–80.6) | 73.1 (69.3–76.6) | 25.7 (20.2–32.1) | 95.2 (92.8–96.8) | - | |
|  | Simplified 2007 IDSA/ATS minor criteria (≥ 3 factors) | 68.9 (57.7–78.3) | 74.9 (71.2–78.3) | 26.3 (20.6–32.9) | 94.9 (92.4–96.6) | 0.79 (0.73–0.84) | |
|  | España SCAP rule (≥ 10 points) | 89.2 (80.1–94.4) | 46.1 (42.0–50.2) | 17.7 (14.2–21.9) | 97.0 (94.3–98.5) | 0.78 (0.73–0.84) | |
|  | PSI (Classes IV and V) | 91.9 (83.4–96.2) | 33.2 (29.5–37.2) | 15.2 (12.2–18.8) | 96.9 (93.5–98.6) | 0.74 (0.68–0.80) | |
|  | CURB-65 (≥ 3 points) | 51.4 (40.2–62.4) | 72.6 (68.8–76.1) | 19.6 (14.6–25.7) | 92.0 (89.1–94.2) | 0.70 (0.64–0.76) | |

|  | **Table of Additional file 4 (*cont.*)** |  |  |  |  |  | |
| --- | --- | --- | --- | --- | --- | --- | --- |
| C. For ICU admission | | | | | | |  |
|  | Rule | Sensitivity,  % (95% CI) | Specificity,  % (95% CI) | PPV,  % (95% CI) | NPV,  % (95% CI) | AUROC  (95% CI) | |
|  | ALL-COP SCORE (≥ 4 points) | 90.9 (81.6–95.8) | 55.1 (51.0–59.1) | 18.8 (14.9–23.5) | 98.2 (96.0–99.2) | 0.83 (0.78–0.87) | |
|  | SMART-COP (≥ 3 points) | 90.9 (81.6–95.8) | 51.5 (47.4–55.5) | 17.7 (14.0–22.1) | 98.0 (95.8–99.1) | 0.77 (0.71–0.83) | |
|  | 2007 IDSA/ATS criteria | 71.2 (59.4–80.7) | 72.1 (68.3–75.6) | 22.6 (17.4–28.8) | 95.6 (93.3–97.2) | 0.80 (0.75–0.86) | |
|  | 2007 IDSA/ATS minor criteria (≥ 3 factors) | 69.7 (57.8–79.5) | 72.3 (68.5–75.8) | 22.3 (17.2–28.5) | 95.4 (93.0–97.0) | - | |
|  | Simplified 2007 IDSA/ATS minor criteria (≥ 3 factors) | 66.7 (54.7–76.8) | 74.0 (70.3–77.4) | 22.7 (17.4–29.1) | 95.1 (92.7–96.7) | 0.78 (0.72–0.83) | |
|  | España SCAP rule (≥ 10 points) | 89.4 (79.7–94.8) | 45.6 (41.6–49.7) | 15.8 (12.5–19.9) | 97.4 (94.8–98.7) | 0.77 (0.71–0.82) | |
|  | PSI (Classes IV and V) | 84.9 (74.3–91.6) | 32.1 (28.4–36.0) | 12.5 (9.8–15.9) | 94.9 (90.8–97.2) | 0.69 (0.62–0.76) | |
|  | CURB-65 (≥ 3 points) | 50.0 (38.3–61.7) | 72.1 (68.3–75.6) | 17.0 (12.4–22.9) | 92.7 (89.9–94.7) | 0.67 (0.60–0.74) | |

Definition of abbreviations:

CI = confidence interval; PPV = positive predictive value; NPV = negative predictive value; AUROC = area under the receiver operating characteristic curve; SMART-COP = systolic blood pressure, multilobar chest x-ray involvement, albumin, respiratory rate, tachycardia, confusion, oxygenation, and arterial PH; SCAP = severe community-acquired pneumoniua; IDSA/ATS = Infectious Disease Society of America/American Thoracic Society; PSI = Pneumonia Severity Index; CURB-65 = confusion, urea level, respiratory rate, blood pressure, and age ≥ 65 yrs.
